# One-year follow-up of chest CT findings in patients after SARS-CoV-2 infection

**DOI:** 10.1186/s12916-021-02056-8

**Published:** 2021-08-09

**Authors:** Yanfei Chen, Cheng Ding, Ling Yu, Wanru Guo, Xuewen Feng, Liang Yu, Junwei Su, Ting Xu, Cheng Ren, Ding Shi, Wenrui Wu, Ping Yi, Jun Liu, Jingjing Tao, Guanjing Lang, Yongtao Li, Min Xu, Jifang Sheng, Lanjuan Li, Kaijin Xu

**Affiliations:** State Key Laboratory for Diagnosis and Treatment of Infectious Diseases, National Clinical Research Center for Infectious Diseases, Collaborative Innovation Center for Diagnosis and Treatment of Infectious Diseases, Department of Infectious Diseases, The First Affiliated Hospital, College of Medicine, Zhejiang University School of Medicine, 79 Qingchun Rd., Hangzhou City, 310003 China

**Keywords:** SARS-CoV-2, Pulmonary radiography, Lung function, Convalescence, Risk factors, Yanfei Chen, Cheng Ding, and Ling Yu contributed equally to the manuscript.

## Abstract

**Background:**

Knowledge about the 1-year outcome of COVID-19 is limited. The aim of this study was to follow-up and evaluate lung abnormalities on serial computed tomography (CT) scans in patients with COVID-19 after hospital discharge.

**Methods:**

A prospective cohort study of patients with COVID-19 from the First Affiliated Hospital, Zhejiang University School of Medicine was conducted, with assessments of chest CT during hospitalization and at 2 weeks, 1 month, 3 months, 6 months, and 1 year after hospital discharge. Risk factors of residual CT opacities and the influence of residual CT abnormalities on pulmonary functions at 1 year were also evaluated.

**Results:**

A total of 41 patients were followed in this study. Gradual recovery after hospital discharge was confirmed by the serial CT scores. Around 47% of the patients showed residual aberration on pulmonary CT with a median CT score of 0 (interquartile range (IQR) of 0–2) at 1 year after discharge, with ground-glass opacity (GGO) with reticular pattern as the major radiologic pattern. Patients with residual radiological abnormalities were older (*p* = 0.01), with higher rate in current smokers (*p* = 0.04), higher rate in hypertensives (*p* = 0.05), lower SaO_2_ (*p* = 0.004), and higher prevalence of secondary bacterial infections during acute phase (*p* = 0.02). Multiple logistic regression analyses indicated that age was a risk factor associated with residual radiological abnormalities (OR 1.08, 95% CI 1.01–1.15, *p* = 0.02). Pulmonary functions of total lung capacity (*p* = 0.008) and residual volume (*p* < 0.001) were reduced in patients with residual CT abnormalities and were negatively correlated with CT scores.

**Conclusion:**

During 1-year follow-up after discharge, COVID-19 survivors showed continuous improvement on chest CT. However, residual lesions could still be observed and correlated with lung volume parameters. The risk of developing residual CT opacities increases with age.

**Supplementary Information:**

The online version contains supplementary material available at 10.1186/s12916-021-02056-8.

## Background

Coronavirus disease 2019 (COVID-19), caused by the severe acute respiratory syndrome coronavirus-2 (SARS-CoV-2), is a global pandemic that has resulted in more than 100 million infections and more than 2 million deaths [1]. According to the research in severe acute respiratory syndrome (SARS) and Middle East respiratory syndrome (MERS), residual radiologic abnormalities and damage of pulmonary functions could last for months or even years [2–5]. In comparison with SARS-CoV-1 and MERS-CoV, COVID-19 seems to be a highly contagious disease with less severity. With millions of worldwide confirmed cases, understanding the recovery dynamics in the COVID-19 discharged patients will be instrumental in recognizing the patient prognosis.

Few studies have analyzed the long-term outcomes after SARS-CoV-2 infection. Residual radiographic abnormalities were observed in a large proportion of COVID-19 survivors at the time of hospital discharge [6]. Three months after the illness onset, a quarter of COVID-19 patients still showed opacities on the chest computed tomography (CT) scans and reduced diffusion capacity [7, 8]. A recent report demonstrated that Wuhan COVID-19 patients mainly presented with fatigue or muscle weakness, sleep difficulties, and anxiety or depression at 6-month follow-up [9]. More than one third of severe COVID-19 survivors demonstrated fibrotic-like changes at 6 months after illness onset [10]. Previous studies have assessed COVID-19 sequela at 1 month, 3 months, and 6 months after hospital discharge [7–10]. Little is known about the 1-year sequela of COVID-19 patients after hospital discharge.

Here, a consecutive cohort of 41 COVID-19 patients was longitudinally followed up for 12 months after discharge. The temporal change of the radiographic features was analyzed. Risk factors of residual CT opacities and the influence of residual CT abnormalities on pulmonary functions at 1 year were also evaluated.

## Methods

### Study design and participants

**Fig. 1:**
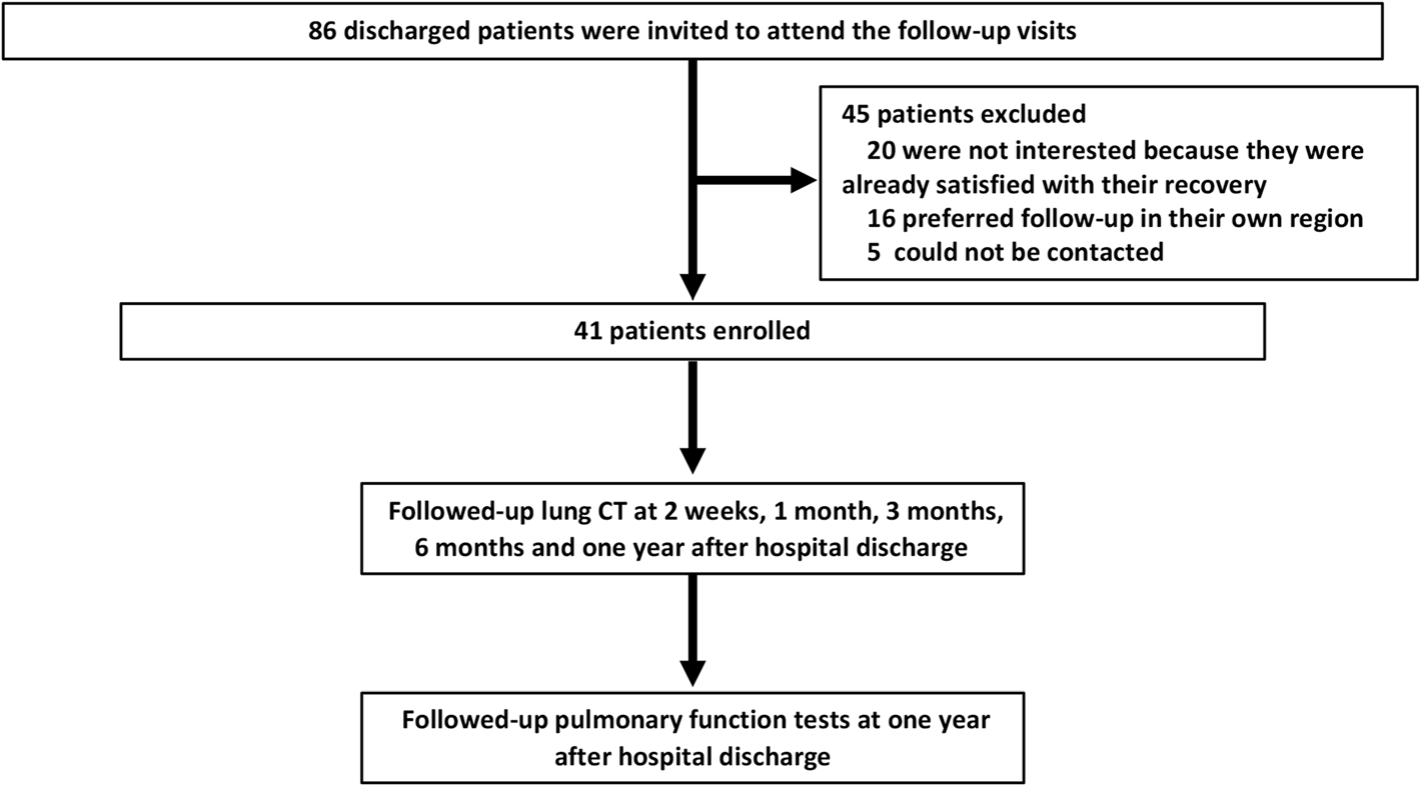
Scheme demonstrating the enrollment of COVID-19 participants and follow-up schedule

This is a prospective longitudinal follow-up study of COVID-19 survivors discharged from the First Affiliated Hospital, Zhejiang University School of Medicine, Hangzhou, China, between February 1 and March 15, 2020. A total of 86 consecutively discharged COVID-19 patients were invited to this follow-up study, and 45 patients were excluded due to the lack of interest or limitation attributed to the patient geographical location (Fig. 1). The chest CT scans of the patients were followed up at 2 weeks, 1 month, 3 months, 6 months, and 1 year after hospital discharge. The COVID-19 patients were categorized based on the disease severity according to Chinese clinical guidance for COVID-19 pneumonia diagnosis and treatment (7th edition) [11]. Briefly, patients could be categorized into four levels of severity: mild, ordinary, severe, and critical illness. Mild illness was defined as mild clinical symptoms without signs of pneumonia on radiologic imaging. Ordinary illness was defined as symptoms of fever, respiratory symptoms, and radiologic manifestation of pneumonia. Severe illness included patients who meet one of the following criteria: respiratory rate ≥ 30 bpm, arterial oxygen saturation (SaO_2_) ≤ 93% at rest, partial pressure of oxygen (PaO_2_)/oxygen absorption concentration (FiO_2_) ≤ 300mmhg, and over 50% lesion progression on chest radiograph within 24–48 h. Critical illness cases are those who meet any of the following criteria: respiratory failure and mechanical ventilation required, occurrence of shock, and complicated with other organ failure requiring intensive care. To simplify the analysis process, mild and ordinary cases were combined as mild group, and severe and critical illness cases were combined as severe group in this study. The ethics approval was obtained from the Institutional Review Board of the First Affiliated Hospital, Zhejiang University School of Medicine (IIT2020-137). Informed written consent was obtained from each participant before enrollment.

### Radiological imaging

Standardized techniques were applied to all radiographic examinations with the same CT equipment. Two radiologists with over 5-year experience independently reviewed the CT images as described previously [12]. Briefly, each lung were divided into three zones: superior (above carina), middle (below carina to the inferior pulmonary vein), and inferior (below the inferior pulmonary vein). Each lung zone (6 lung zones in total) was scored according to the following rules: 0, 0% involvement; 1, less than 25% involvement; 2, 25% to 50% involvement; 3, 50% to 75% involvement; and 4, 75% involvement or higher. The sum of the total scores provided the overall lung involvement (the maximum value for both lungs was 24) [12].

The predominant pattern on CT scans were categorized as (1) pure ground-glass opacity (GGO), which was defined as increased lung density with no obscuration of the underlying lung marks; (2) reticular pattern, which was defined as GGO with reticulation, includes fibrous cord shadow, subpleural line, and the presence of bronchiectasis; (3) GGO with consolidation, which was defined as increased lung density with obscuration of the underlying lung marks; and (4) mixed pattern, which meant combination of consolidation, GGO, and reticular opacities. The corresponding figures of these radiologic patterns are listed in Additional file 1: Figure S1. The CT scans obtained during hospitalization and two weeks after hospital discharge were marked with the time since the illness onset. CT scans obtained at 1st, 3rd, 6th, and 12th month after hospital discharge, which were time-marked with the time since hospital discharge. In total, 317 chest CTs were analyzed from 41 patients. Each patient had an average of 7.7 ± 1.5 CTs (range 4–10 CTs).

### Pulmonary function tests (PFTs)

PFTs were performed at 12 months after discharge. Parameters of total lung capacity (TLC), residual volume (RV), forced vital capacity (FVC), forced expiratory volume in 1 s (FEV1), FEV1/FVC ratio, peak expiratory flow (PEF), and diffusing capacity for carbon monoxide (DLCO) were measured using the Sensor Medic Vmax System (USA) according to the standard protocols. The lung function parameters were expressed as the percentage of predicted normal values, except for PEF and FEV1/FVC [13].

### Statistical analysis

Demographic characteristics were expressed either as median and interquartile range (IQR) or as absolute values along with percentages for categorical variables. Continuous variables were tested by Kruskal-Wallis test for the inter-group comparisons. Categorical variables among the different groups, as well as the clinical characteristics between mild and severe groups, were tested with Fisher’s exact test. To identify independent risk factors associated with residual CT legions, potential influencing factors with p values < 0.1 by univariate analyses were further analyzed by multiple logistic regressions. Spearman's rank correlation coefficient was used to evaluate the correlations between CT scores and PFTs index. All statistical analyses were performed by using the SAS 9.4 software (SAS Institute Inc., Cary, NC, USA). The significance level of the hypothesis tests was set at 0.05 (two-sided).

## Results

### Clinical characteristics and 1-year PFTs of the patients

Clinical characteristics of these patients are summarized in Table 1. Among the patients enrolled, there were 25 patients with mild illness and 16 patients with severe or critical illness. The severe group had higher ratio of males (*p* = 0.018), as well as higher occurrence of ICU admissions (*p* = 0.024), treatment of high flow nasal cannula (HFNC) (*p* = 0.001), and corticosteroid use (*p* = 0.017). Overall, lung functions were well preserved after 1-year recovery (Table 1). Upon 1-year follow-up, 37 patients (92.7%) had FEV1/FVC ratio > 70%. Only 3 patients (7.3%) had a pulmonary diffusion abnormality (DLCO < 80% predicted), and five patients (12.2%) showed a TLC below 80%. Comparing the PFTs between the mild group and severe group (Table 1), median value of RV was slightly lower in the severe group (median 109.5, IQR 105.5–120.5) than the mild group (median 131, IQR 110–144) (*p* = 0.014). Non-significant reduction of TLC was observed in the severe group than that in the mild group (*p* = 0.057). Other parameters of PFTs, such as FEV1/FVC ratio, FVC, FEV1, PEF, and DLCO, were comparable between the mild and severe groups.

**Table 1:** Clinical characteristics of the patients

	All (***n*** = 41)	Mild patients (***n*** = 25)	Severe patients (***n*** = 16)	p value
Age, years	51 (38, 59)	48 (38, 58)	51.5 (37, 60.5)	0.698
Male, n (%)	24 (58.5%)	11 (44%)	13 (81.2%)	**0.018**
BMI, kg/m^2^	24.2 (21.9, 25.1)	24.2 (21.9, 25.1)	24.0 (22.2, 25.8)	0.873
Smoking history	4 (9.76%)	2 (8%)	2 (12.5%)	0.636
*Comorbidities*				
Hypertension, n (%)	10 (24.4%)	4 (16%)	6 (37.5%)	0.118
Type 2 diabetes, n (%)	4 (9.7%)	2 (8%)	2 (12.5%)	0.636
Coronary artery heart disease, n (%)	2 (4.9%)	1 (4%)	1 (6.25%)	0.745
COPD/emphysema, n (%)	2 (4.9%)	2 (8%)	0 (0%)	0.246
*Illness progress*				
Duration from illness onset to hospital admission, days	5 (2, 7)	6 (4, 8)	5 (1.5, 6)	0.427
Nasopharyngeal viral RNA shedding duration, days	19 (15, 26)	17 (13, 23)	22 (16, 28)	0.077
Duration of hospitalization, days	17 (14, 23)	16 (12, 19)	19 (15.5, 25.5)	0.071
ICU admission, n (%)	3 (7.3%)	0 (0%)	3 (18.7%)	**0.024**
HFNC, n (%)	6 (14.6%)	0 (0%)	6 (37.5%)	**0.001**
Corticosteroid treatment, n (%)	30 (73.2%)	15 (60%)	15 (93.75%)	**0.017**
Median corticosteroid dose	60 (40,80)	40 (40, 60)	80 (40,80)	0.112
*PFTs at 1-year follow-up*^*#*^				
DLCO < 80%, n (%)	3 (7.3%)	1 (4.0%)	2 (12.5%)	0.515
TLC < 80%, n (%)	5 (12.2%)	2 (8%)	3 (18.7%)	0.538
FEV1/FVC < 0.70, n (%)	3 (7.3%)	3 (12.0%)	0 (0%)	0.087
FVC %	97 (86, 105)	99 (93, 106)	91.5 (81, 103.5)	0.25
FEV1 %	100 (87, 104)	100 (91, 104)	95.5 (80, 104.5)	0.399
PEF	88 (71, 100)	92 (69, 100)	82.5 (71, 96.5)	0.584
FEV1/FVC	82.4 (78.3, 85.1)	83 (77.9, 85)	81.45 (78.3, 85.6)	0.947
DLCO %	112 (99, 121)	113 (106, 122)	101 (91.5, 120)	0.067
TLC %	96 (88, 107)	99 (94, 110)	92.5 (86, 104)	0.057
RV %	120 (106, 132)	131 (110, 144)	109.5 (105.5, 120.5)	**0.014**

The quantitative data are shown as median data and inter quartile range data in brackets

The occurrence data are shown as no. (%). Values indicate no. of positive results/total no. of patients with available assay results

Kruskal-Wallis test for continuous variables or Fisher’s exact test for categorical variables was used for comparison between groups when applicable with *p* < 0.05 as significant

*Abbreviations*: BMI, body mass index; ICU, intensive care unit; HFNC, high flow nasal catheter oxygen therapy; PFTs, pulmonary function tests; FVC, forced vital capacity; FEV1, forced expiratory volume in the first 1 s of expiration; PEF, peak expiratory flow; DLCO, diffusing capacity of the lung for carbon monoxide; TLC, total lung capacity; RV, residual volume

^#^Pulmonary function tests were expressed as percent of the predicted value

### Longitudinal changes of chest CT after SARS-CoV-2 infection

**Fig. 2:**
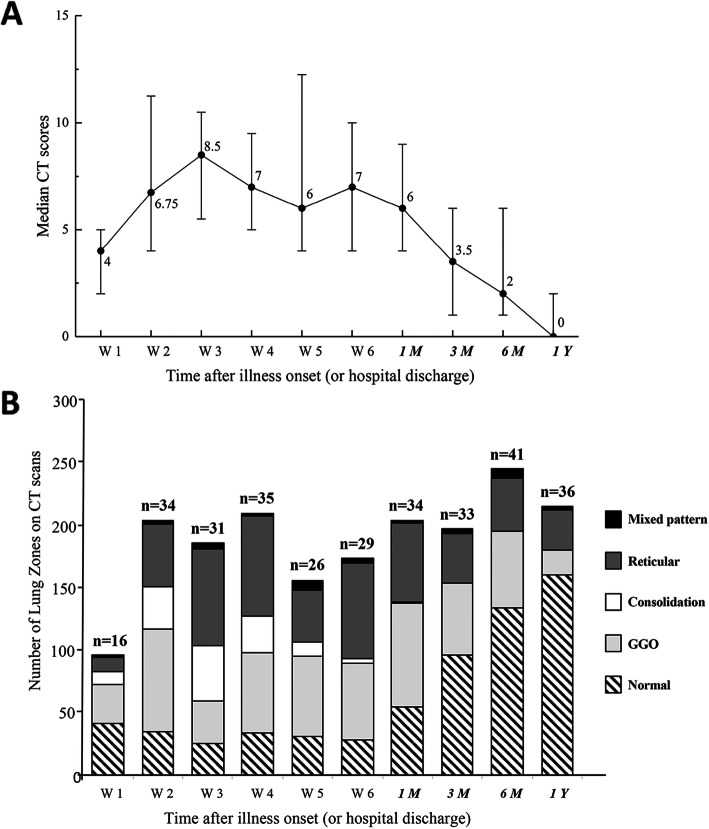
**A** Line graph demonstrating the median CT scores at various time points in weeks after symptoms onset. **B** Stacked-bar graph showing the distribution of different patterns of lung changes on CT scans at various time points from the onset of symptoms. Black color indicates bronchiectasis, dark gray indicates reticular pattern, white indicates consolidation, light gray indicates ground-glass opacities, and striped indicates normal. The time points in italics indicate the time after hospital discharge (1M, 3M, 6M, and 1Y indicated 1 month, 3 months, 6 months, and 1 year after hospital discharge, respectively). The number of patients at each sampling point was listed on the top of the bar

**Fig. 3:**
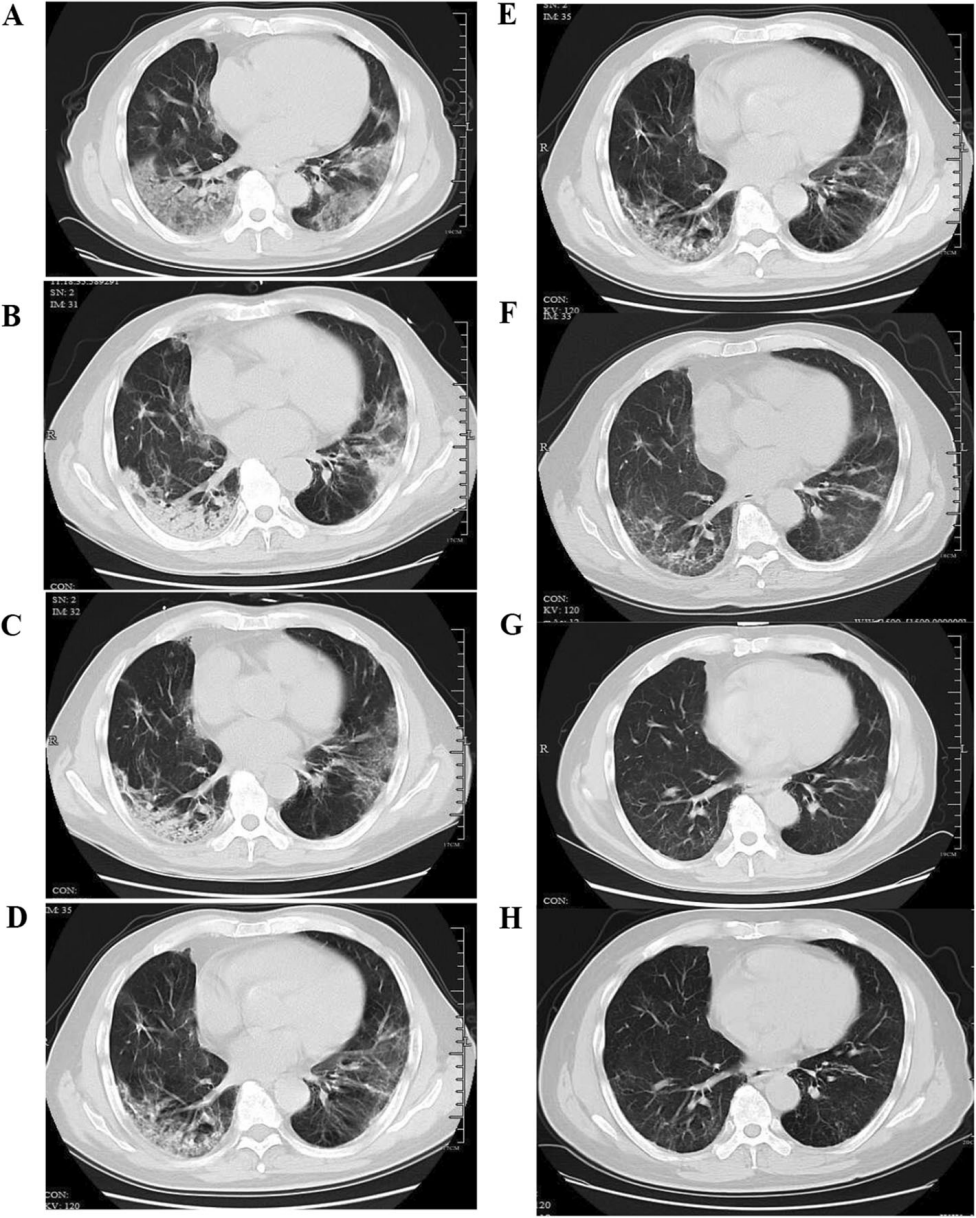
Transverse CT scans of a 72-year-old man with severe COVID-19. He was presented with fever and cough on January 29, 2020, confirmed with SARS-CoV-2 infection on February 1, then admitted to the hospital on the same day, and was treated with corticosteroid and high flow nasal cannula during hospitalization. **A** Scan obtained on day 7 showing diffuse subpleural GGO. **B** Scan obtained on day 14 showing subpleural GGO with partial consolidation. **C** Scan obtained on day 21 of illness showing subpleural GGO, and consolidation partly absorbed. **D** Scan obtained on day 28 of illness showing irregular linear opacities that developed in the areas of GGO. **E** Scan obtained on 1 month after discharge (day 62 of illness) demonstrating irregular linear opacities and subpleural reticulation. **F** Scan obtained on 3 months after discharge (day 112 of illness) showing subpleural reticulation. **G** Scan obtained at 6 months after discharge (day 210 of illness) showing slight irregular linear opacities and reticulation on the basis of GGO. **H** Scan obtained at 1 year after discharge (day 390 of illness) showing subtle irregular linear opacities and reticulation on the basis of GGO

There was a gradual recovery process after hospital discharge as confirmed by the serial CT scores at 1-year (Fig. 2A). The extent of disease showed a marked increase during the first two weeks after onset of symptoms and peaked in the third week with a median CT score of 8.5 (IQR 5.5–10.5), followed by a plateau phase until 1 month after hospital discharge. The recovery rate accelerated 1 month after hospital discharge. Upon 1 year after discharge, 47% of the patients were observed with subtle residual opacities (median 0, IQR 0–2). Dynamic changes of CT in a 72-year-old man with SARS-CoV-2 infection, who received treatment of corticosteroid and HFNC during hospitalization, are shown in Fig. 3. As can be seen, bilateral diffuse subpleural GGO with partial consolidation was gradually absorbed. Subpleural line and reticulation, irregular linear opacities developed in the areas of GGO approximately 1 month after discharge, and slight irregular linear opacities could still be noted at 1 year after discharge.

Moreover, we found that the predominant pattern on CT scans changed over time (Fig. 2B). Specifically, the extent of consolidation peaked (40.2%) at the 3rd week after onset of symptoms and decreased thereafter. Pure GGO or GGO with reticular pattern were the most common abnormal patterns since onset of symptoms until 12 months after hospital discharge. The ratio of lung zones with normal CT findings increased from 27.0% at 1 month, to 48.5% at 3 months, 54.5% at 6 months, and 65.4% at 1 year after hospital discharge (Additional file 1: Table S1). By the end of 1-year follow-up, only 13.0% of the lung zones had signs of GGO with reticular pattern, as well as 8.1% with pure GGO (Additional file 1: Table S1).

**Fig. 4:**
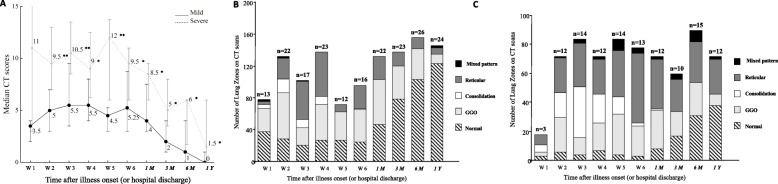
**A** Line graph demonstrating the median CT scores of mild and severe patients at various time points after onset of symptoms. Solid line mild patients; dotted line severe patients. **B** Stacked-bar graph showing the distribution of different patterns of lung changes in mild patients on CT scans at various time points. **C** Stacked-bar graph showing the distribution of different patterns of lung changes in severe patients on CT scans at various time points. Black color indicates bronchiectasis, dark gray indicates reticular pattern, white indicates consolidation, light gray indicates the ground-glass opacities, and striped indicates normal. The time points in italics were time after hospital discharge (1m, 3m, and 6m indicated 1 month, 3 months, and 6 months after hospital discharge, respectively). The number of patients at each sampling point was listed on the top of the bar

The comparison of dynamic changes in CT scores at various time points between mild and severe patients is plotted in Fig. 4A. At 1 year after discharge, the median score of residual abnormalities was significantly lower in the mild group (median 0, IQR 0–1) compared to the severe group (median 1.5; IQR 1–6) (*p* = 0.03). The CT scores of severe patients peaked early after illness onset and maintained at high levels for several weeks before a slow decline. For mild patients, the radiographic abnormalities gradually reached its peak at the 3rd week after illness onset. And the peak score was lower than that in the severe group (*p* = 0.002). CT scores of the severe group were higher than those of the mild group for all the time-points except for the first week during the observation period (p < 0.05, Fig. 4A). Both the mild and severe groups showed slow recovery during the early recovery phases (from the 3rd week to the 6th week after illness onset). Higher prevalence of consolidation was observed in the severe group during acute phase than that in the mild group (Fig. 4B, C, Table S1). For the patients with mild illness during acute phase, the ratio of lung zones with normal CT findings increased from 35.6% at 1 month, to 57.2% at 3 months, 66% at 6 months, and 78.8% at 1 year after hospital discharge (Fig. 4B, Table S1). Similar increasing trend of normal scans could also be noted in the severe group, i.e.,11.1% at 1 month, 28.3% at 3 months, 34.4% at 6 months, and 42.2% at 1 year (Fig. 4B, Table S1).

### Risk factors for residual radiological abnormalities at 1 year after discharge

Patients were divided into two groups according to the presence (*n* = 17) or absence (*n* = 19) of residual lesions on CT at 1 year after discharge. Epidemic and clinical characteristics were compared between the two subgroups (Table 2). Patients with residual radiological abnormalities were older (*p* = 0.01), with higher occurrence in current smokers (*p* = 0.04), hypertensives (*p* = 0.05), lower SaO_2_ (*p* = 0.004), and secondary bacterial infections during acute phase (*p* = 0.02). Multivariable logistic regression analysis indicated that age (OR 1.08, 95% CI 1.01–1.15, *p* = 0.02) was a risk factor associated with residual radiological abnormalities at 1 year after discharge (Table 3).

**Table 2:** Comparison of clinical characteristics between groups with or without residual CT abnormalities

	No residual legions	Presence of residual legions	
	(***n*** = 19)	(***n*** = 17)	p value
Age, years	40 (34, 54)	55 (48, 62)	**0.014**
Male, n (%)	22.88 (21.51, 25.15)	24.24 (23.42, 25.24)	0.318
BMI, kg/m^2^	11 (57.9%)	10 (58.8%)	1
Drinking history, n (%)	0 (0%)	1 (5.9%)	0.472
Current smoker, n (%)	0 (0%)	4 (23.5%)	**0.04**
Hypertension, n (%)	2 (10.5%)	7 (41.2%)	**0.05**
Type 2 diabetes, n (%)	1 (5.3%)	3 (17.6%)	0.325
COPD/emphysema, n (%)	1 (5.3%)	1 (5.9%)	0.933
Duration from illness onset to hospital admission, days	5 (2, 8)	6 (4, 7)	0.962
Severe illness during hospitalization	4 (21.1%)	8 (47.1%)	0.158
Nasopharyngeal viral RNA shedding duration, days	15 (12, 23)	19 (13, 21)	0.515
Peak CT score during hospitalization	6.5 (4, 9.5)	9 (6, 13)	0.301
Corticosteroid treatment, n (%)	11 (57.9%)	13 (76.5%)	0.302
Secondary bacterial infection, n (%)	0 (0%)	5 (29.4%)	**0.016**
ICU admission, n (%)	1 (5.3%)	2 (11.8%)	0.593
HFNC, n (%)	2 (10.5%)	4 (23.5%)	0.391
*Laboratory indicators on admission*			
Lymphocyte count (* 10^9^/L)	0.9 (0.6, 1.4)	0.9 (0.5, 1.1)	0.633
D-dimer, mg/L	318 (170, 425)	328 (178, 477)	0.666
ALT, U/L	26 (19, 53)	22 (15, 27)	0.109
AST, U/L	22 (18, 41)	19 (17, 21)	0.089
CRP, mg/L	10.33 (3.55, 27.87)	20.94 (11.39, 68.3)	0.096
IL-6, pg/mL	13.58 (5.63, 50.46)	14.27 (5.29, 24.01)	0.763
ESR, mm/h	27 (8, 42)	27 (13, 74)	0.31
SaO_2_	98.5% (97.6%, 99.6%)	96.9% (93.7%, 98.3%)	**0.004**
*PFTs at 1-year follow-up*^*#*^			
FVC %	100 (94, 106)	95 (84, 102)	0.096
FEV1 %	100 (93, 103)	98 (87, 105)	0.579
PEF	95 (69, 105)	82 (69, 95)	0.168
FEV1/FVC	82.3 (77.9, 85.1)	82.4 (78.3, 84.7)	0.987
DLCO %	113 (105, 124)	112 (93, 121)	0.375
TLC %	107 (96, 111)	94 (83, 99)	**0.008**
RV %	132 (121, 144)	105 (95, 120)	**< .0001**

Kruskal-Wallis test for continuous variables or Fisher’s exact test for categorical variables was used for comparison between groups when applicable with *p* < 0.05 as significant

The occurrence data are shown as no. (%) unless otherwise indicated. Values indicate no. of positive results/total no. of patients with available assay results

The time data are shown as median data and inter quartile range data in brackets

^#^Pulmonary function tests were expressed as percent of the predicted value

*Abbreviations*: BMI, body mass index; COPD, chronic obstructive pulmonary disease; ICU, intensive care unit; ALT, Alanine aminotransferase. AST, Aspartate aminotransferase; ESR, Erythrocyte sedimentation rate; CRP, C-reaction protein; IL-6, interleukin-6; SaO_2_, oxygen saturation; HFNC, high flow nasal catheter oxygen therapy; PFTs, pulmonary function tests; FVC, forced vital capacity; FEV1, forced expiratory volume in the first 1 s of expiration; PEF, peak expiratory flow; DLCO, diffusing capacity of the lung for carbon monoxide; TLC, total lung capacity; RV, residual volume

**Table 3:** Multivariable analyses of factors associated with CT residual abnormalities

	Multivariable analysis			Stepwise analysis		
Variable	Odds ratio (OR)	95% CI	p value	Odds ratio (OR)	95% CI	p value
Age	1.073	1.009–1.140	0.024	1.080	1.014–1.150	0.017
Current smoking	12.994	0.460–367.1	0.132			
Hypertension	5.000	0.915–27.3	0.063			
Secondary bacterial infection	17.158	0.664–443.5	0.087			

### Correlations of radiological residual abnormalities and PFTs

**Fig. 5:**
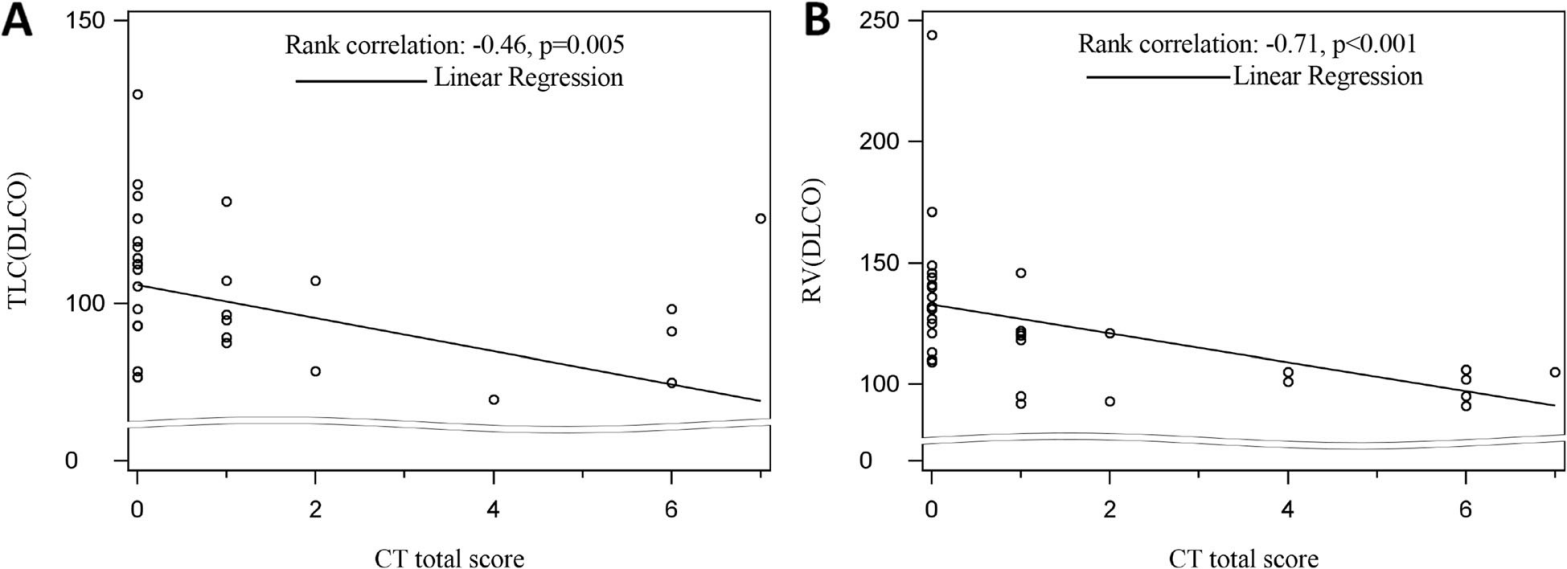
**A** Scatter-plot showing significant correlation between CT score and total lung capacity (TLC) at 1 year after hospital discharge. **B** Scatter-plot showing significant correlation between CT score and residual volume (RV) at 1 year after hospital discharge

Despite no significant difference of most of the parameters of PFTs, such as FVC, FEV1, PEF, DLCO, and FEV1/FVC ratio, lung volume parameters of TLC (*p* = 0.008) and RV were significantly lower (*p* < 0.001) in the group with residual CT abnormalities than that in the group without abnormalities (Table 2). The residual CT lesion scores were negatively correlated with TLC scores at 1 year (R = − 0.46; *p* = 0.005), as well as the value of RV (R = − 0.71; *p* < 0.001) (Fig. 5A, B).

## Discussion

Existing knowledge regarding the longitudinal changes as well as long-term outcomes of COVID-19 remains limited [9]. Here, we reported a 1-year follow-up study of 41 COVID-19 patients by chest CTs and PFTs. To our knowledge, our study provided the longest follow-up interval as well as the longitudinal description of CT series of COVID-19 patients. We found that age (*p* = 0.01), smoking (*p* = 0.04), hypertension (*p* = 0.05), lower SaO_2_ (*p* = 0.004), and secondary bacterial infections during acute phase (*p* = 0.02) were significantly associated with residual radiological abnormalities. In addition, we observed that lung volume parameters of TLC (*p* = 0.008) and RV (*p* < 0.001) were significantly lower (*p* < 0.001) in patients with residual CT abnormalities than those without abnormalities at 1 year after hospital discharge.

Data were limited about the 1-year radiologic sequela. Our results indicated substantial recovery on chest CTs occurred at 1 year after discharge with a median CT score of 0 and 65.4% of the lung zones as normal. Tang et al. reported a case of 55-year-old woman returned to normal lung parenchyma by the time of 1 year after illness onset [14]. The serial CTs in our cohort also provided the longitudinal lung changes in COVID-19 patients in the early and late convalescent periods. The recovery rate was much slower during the early convalescent phase (within 1 month after discharge) than late convalescent phase (from 1 month to 1 year after discharge). A slow decline of CT scores has been documented in early convalescence of COVID-19 patients (within 30 days after illness onset) previously [15]. The possible reason of slow recovery might be related to corticosteroid use. Most patients in our cohort underwent corticosteroid treatment which was weaned off and stopped in the first month after discharge. Whether the use of corticosteroid could lead to delay of the recovery on CT scans needs to be verified in future studies with larger sample size. In the previous 15-year follow-up study with serial CT scans in SARS infected patients, lesion absorption and recovery occurred to a greater extent within the first 12 months after infection, and then remained stable in the following 15 years [16]. Thus, how long the residual opacities would persist after COVID-19 warrants further research with longer follow-up in the future.

The development of pulmonary fibrosis is an important sequela in patients after severe respiratory infections [17]. Since the observation of substantial fibrotic consequences following infection of SARS-CoV-1 and MERS-CoV [18–20], concern has been raised about the prevalence and persistence of lung fibrosis after COVID-19. Based on previous data, lung fibrotic-like changes (the presence of traction bronchiectasis, parenchymal bands, and honeycombing) were observed in 35% of the patients who recovered from severe COVID-19 at 6 months after illness onset [10]. In this study, GGO with reticular pattern was observed in 31% of the lung zones in severe patients at 6 months, which is comparable with the previous reports. This value declined to 26.7% for the severe patients and 13.0% for the whole cohort by the time of 12 months after discharge, showing a gradual recovery on lung radiology. However, it remains uncertain whether the reticulation changes observed here represents true fibrotic changes. Further studies with longer follow-up intervals are still needed.

Patients with residual lesions on CT scans at 1 year after discharge were older. Besides, age was identified as a predictor of residual radiologic opacities. Song et al. reported older (> 50 years) COVID-19 patients had more consolidation lesions than younger patients [21]. Wang et al. found that the mass of pulmonary involvement of older patients (> 45 years) was significantly more severe and peaked later than that of younger patients (≤ 45 years) [22]. At 3 months after discharge, age was associated with the presence of GGO [8]. In the previous 6-month follow-up study, old age (> 50 years old) was identified as the independent risk factor of fibrotic-like changes after SARS-CoV-2 infection [10]. The severity and outcome of COVID-19 is closely associated with the age of patients [23, 24]. Why the aged COVID-19 patients tend to be sicker and recover slower is not yet known. The potential molecular mechanisms might include epigenetic dysregulation of angiotensin-converting enzyme 2 and hyperactivation of NOD-like receptor protein 3 as a trigger of cytokine storms in aged people [25]. Also, age-related differences regarding COVID-19 disease severity is suggested to be related to negative associations with CD8+ T cell count and positive associations with inflammatory responses and liver damage [26]. Another possible explanation is the increased incidence of comorbidities with age, including hypertension, coronary heart disease, chronic obstructive pulmonary disease, and diabetes [27]. These comorbidity diseases and the corresponding medications would influence the illness process. Further studies with larger sample size are warranted to stratify the effect of comorbidities.

The diffusion capacity was the most influenced pulmonary function in post-infection COVID-19 patients at early recovery phase [28]. There was a considerable proportion (22–56% across different severity scales) of COVID-19 patients that had a pulmonary diffusion abnormality 6 months after symptom onset [29]. Our results demonstrated that only 7.3% of the patients showed DLCO below 80% at the time of 1 year after discharge, which suggests good recovery of diffusion capacity from 6 months to 12 months. The pulmonary restriction abnormality has also been noted in early convalescence [28]. At the time of 6 months after symptom onset, decreased RV was observed in patients between scale 3 (not requiring supplemental oxygen) and scale 5-6 (requiring HFNC and mechanical ventilation) [29]. In our study, the value of TLC and RV in participants with CT residual lesions was much less than that of participants without residual lesions. Furthermore, significant negative correlations were observed between residual opacities and PFTs of TLC and RV (Fig. 5). Taken together, it was proposed that considerable impairment of lung volume, especially in patients with CT abnormalities. The results here are consistent with histological features of SARS-CoV-1 cases, which include diffuse alveolar damage in the early phase of the disease and dense septal and alveolar fibrosis in the later course of disease [30]. However, since the level of TLC and RV in both groups (with or without residual CT opacities) was generally normal, the reductions might have no clinical implications.

There are a few limitations to our study. Firstly, the baseline CT scans prior to the illness onset is unavailable; thus, it is difficult to compare the results before and after the illness. However, the proportion of patients with chronic respiratory in this study is fairly low, so it should be acceptable to assume that the baseline CT scans for majority of patients would be normal. Secondly, the sample size in our study is relatively small, and our findings including the identified risk factors should be confirmed by observations conducted at multiple centers and with larger sample sizes. Thirdly, subtle residual radiological opacities were observed in patients at 1-year follow-up. When the remaining radiological abnormalities completely resolve needs to be investigated in further studies. Finally, deep learning-based image processing software are showing great potential in quantitatively analyzing chest CT images [31], which could be used in further studies to provide more quantitative data (such as effective lung volume, lesion volume).

## Conclusion

In conclusion, COVID-19 survivors showed continuous improvement on lung CT scans during 1-year recovery. Residual lesions could still be observed in pulmonary radiography and correlated with lung volume parameters. Old patients are at high risk of developing residual CT abnormalities. Our study offers a comprehensive understanding of the longitudinal lung changes in COVID-19 patients during the acute and convalescent periods, which could help in providing theoretical basis for rehabilitation.

## Supplementary Information

**Additional file 1 Table S1**: Distribution of residual abnormalities patterns at different time points. **Figure S1**: Radiologic patterns observed in follow-up chest CTs of patients recovered from COVID-19.

## Data Availability

The datasets during and/or analyzed during the current study available from the corresponding author on reasonable request.

## Abbrevations

COVID-19: Coronavirus disease 2019
SARS: Severe acute respiratory syndrome
MERS: Middle East respiratory syndrome
CT: Computed topography
GGO: Ground-glass opacity
ALT: Alanine aminotransferase
AST: Aspartate aminotransferase
ESR: Erythrocyte sedimentation rate
CRP: C-reaction protein
IL-6: Interleukin-6
SaO_2_: Oxygen saturation
PFTs: Pulmonary function tests
TLC: Total lung capacity
RV: Residual volume
FVC: Forced vital capacity
FEV1: Forced expiratory volume in 1 s
PEF: Peak expiratory flow
DLCO: Diffusing capacity for carbon monoxide
IQR: Interquartile range
HFNC: High flow nasal cannula
