# Supplementary material for: One-year follow-up of chest CT findings in patients after SARS-CoV-2 infection
Source: BMC Med. 2021 Aug 9;19:191. doi: 10.1186/s12916-021-02056-8 (PMC8349604; doi:10.1186/s12916-021-02056-8)

Additional Files

| **Table S1. Distribution of residual abnormalities patterns at different timepoints** | | | | | | | | | | | | | | | | | | | | | |
| --- | --- | --- | --- | --- | --- | --- | --- | --- | --- | --- | --- | --- | --- | --- | --- | --- | --- | --- | --- | --- | --- |
|  |  | | **Week1** | | **Week2** | | **Week3** | | **Week4** | | **Week5** | | **Week6** | | **1 month** | | **3 months** | | **6 months** | | **1 year** |
|  | **Number of patients** | | 16 | | 34 | | 31 | | 35 | | 26 | | 29 | | 34 | | 33 | | 41 | | 36 |
|  | **Number of lung zones** | | 96 | | 204 | | 186 | | 210 | | 156 | | 174 | | 204 | | 198 | | 246 | | 216 |
|  | **Normal** | | 41 (42.7%) | | 35 (17.2%) | | 25 (13.4%) | | 34 (16.2%) | | 31 (19.9%) | | 28 (16.1%) | | 55 (27.0%) | | 96 (48.5%) | | 134 (54.5%) | | 161 (65.4%) |
|  | **GGO** | | 32 (33.3%) | | 82 (40.2%) | | 34 (18.3%) | | 64 (30.5%) | | 64 (41.0%) | | 62 (35.6%) | | 83 (40.7%) | | 58 (29.3%) | | 62 (25.2%) | | 20 (8.1%) |
| **The whole patientcohort** | **Consolidation** | | 10 (10.4%) | | 34 (16.7%) | | 45 (24.2%) | | 30 (14.3%) | | 12 (7.7%) | | 3 (1.7%) | | 1 (0.5%) | | 0 (0.0%) | | 0 (0.0%) | | 0 (0.0%) |
|  | **Reticula pattern** | | 11 (11.5%) | | 50 (24.5%) | | 78 (41.9%) | | 80 (38.1%) | | 41 (26.3%) | | 77 (44.3%) | | 63 (30.9%) | | 40 (20.2%) | | 42 (17.1%) | | 32 (13.0%) |
|  | **Mixed** | | 2 (2.1%) | | 3 (1.5%) | | 4 (2.2%) | | 2 (1.0%) | | 8 (5.1%) | | 4 (2.3%) | | 2 (1.0%) | | 4 (2.0%) | | 8 (3.3%) | | 3 (1.2%) |
|  | **Number of patients** | | 13 | | 22 | | 17 | | 23 | | 12 | | 16 | | 22 | | 23 | | 26 | | 24 |
|  | **Number of lung zones** | | 78 | | 132 | | 102 | | 138 | | 72 | | 96 | | 132 | | 138 | | 156 | | 144 |
|  | **Normal** | | 38 (48.7%) | | 29 (22.0%) | | 21 (20.6%) | | 27 (19.6%) | | 27 (37.5%) | | 25 (26.0%) | | 47 (35.6%) | | 79 (57.2%) | | 103 (66.0%) | | 123 (78.8%) |
|  | **GGO** | 29 (37.2%) | | 58 (43.9%) | | 22 (21.6%) | | 45 (32.6%) | | 36 (50.0%) | | 41 (42.7%) | | 56 (42.4%) | | 41 (29.7%) | | 39 (25.0%) | | 12 (7.7%) | |
| **Mild patient cohort** | **Consolidation** | | 5 (6.4%) | | 17 (12.9%) | | 10 (9.8%) | | 10 (7.2%) | | 0 (0.0%) | | 1 (1.0%) | | 0 (0.0%) | | 0 (0.0%) | | 0 (0.0%) | | 0 (0.0%) |
|  | **Reticula pattern** | | 4 (5.1%) | | 26 (19.7%) | | 48 (47.1%) | | 56 (40.6%) | | 9 (12.5%) | | 29 (30.2%) | | 29 (22.0%) | | 18 (13.0%) | | 14 (9.0%) | | 8 (5.1%) |
|  | **Mixed** | | 2 (2.6%) | | 2 (1.5%) | | 1 (1.0%) | | 0 (0.0%) | | 0 (0.0%) | | 0 (0.0%) | | 0 (0.0%) | | 0 (0.0%) | | 0 (0.0%) | | 3 (1.9%) |
|  | **Number of patients** | | 3 | | 12 | | 14 | | 12 | | 14 | | 13 | | 12 | | 10 | | 15 | | 12 |
|  | **Number of lung zones** | | 18 | | 72 | | 84 | | 72 | | 84 | | 78 | | 72 | | 60 | | 90 | | 72 |
|  | **Normal** | | 3 (16.7%) | | 6 (8.3%) | | 4 (4.8%) | | 7 (9.7%) | | 4 (4.8%) | | 3 (3.8%) | | 8 (11.1%) | | 17 (28.3%) | | 31 (34.4%) | | 38 (42.2%) |
|  | **GGO** | | 3 (16.7%) | | 24 (33.3%) | | 12 (14.3%) | | 19 (26.4%) | | 28 (33.3%) | | 21 (26.9%) | | 27 (37.5%) | | 17 (28.3%) | | 23 (25.6%) | | 8 (8.9%) |
| **Severe patient cohort** | **Consolidation** | | 5 (27.8%) | | 17 (23.6%) | | 35 (41.7%) | | 20 (27.8%) | | 12 (14.3%) | | 2 (2.6%) | | 1 (1.4%) | | 0 (0.0%) | | 0 (0.0%) | | 0 (0.0%) |
|  | **Reticula pattern** | | 7 (38.9%) | | 24 (33.3%) | | 30 (35.7%) | | 24 (33.3%) | | 32 (38.1%) | | 48 (61.5%) | | 34 (47.2%) | | 22 (36.7%) | | 28 (31.1%) | | 24 (26.7%) |
|  | **Mixed** | | 0 (0.0%) | | 1 (1.4%) | | 3 (3.6%) | | 2 (2.8%) | | 8 (9.5%) | | 4 (5.1%) | | 2 (2.8%) | | 4 (6.7%) | | 8 (8.9%) | | 2 (2.2%) |
| The occurrence data are shown as no. (%) unless otherwise indicated. Values indicate no. of positive results/total no. of lung areas. | | | | | | | | | | | | | | | | | | | | | |
| Each lung was divided into three zones: superior (above carina), middle (below carina to the inferior pulmonary vein) and inferior (below the inferior pulmonary vein). Each lung area (6 lung zones in total) was estimated with the main abnormality pattern. | | | | | | | | | | | | | | | | | | | | | |

Figure S1. Radiologic patterns observed in follow-up chest CTs of patients recovered from COVID-19.


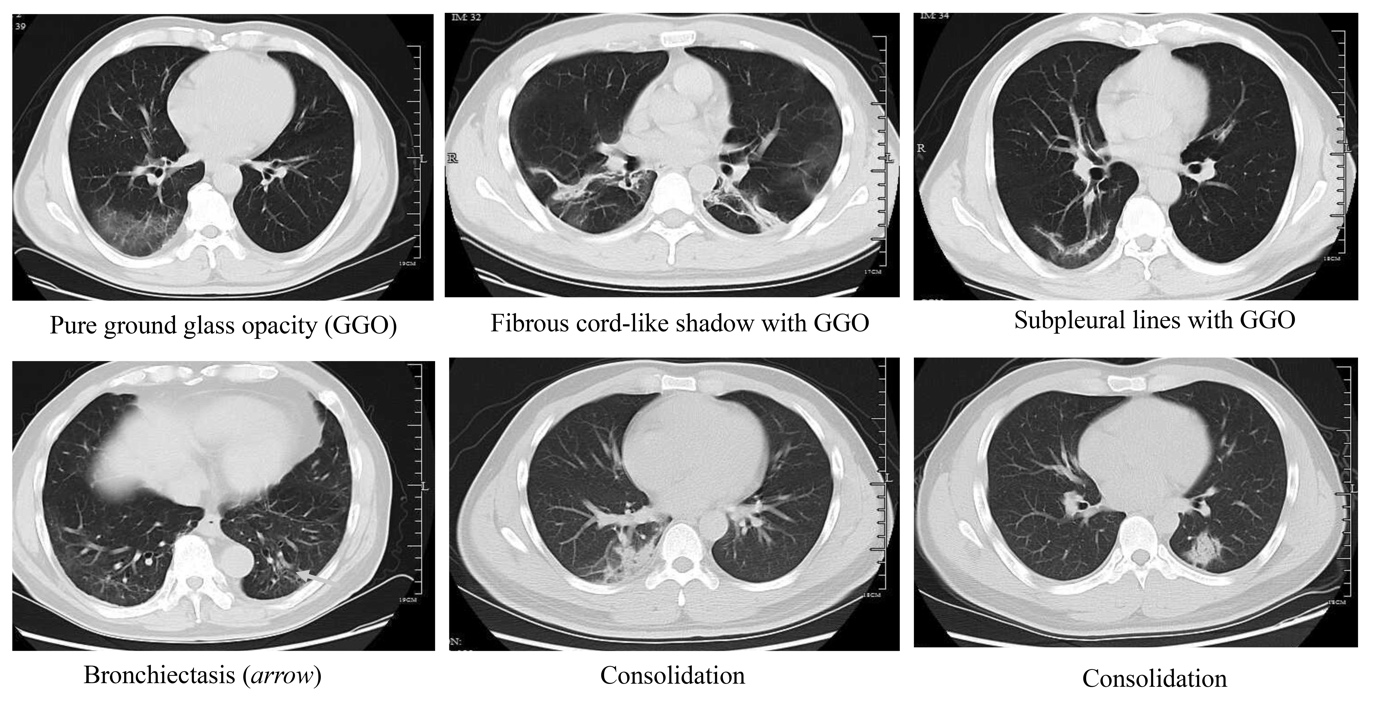

Supplement: Supplementary file 1 — Additional file 1 Table S1: Distribution of residual abnormalities patterns at different time points. Figure S1: Radiologic patterns observed in follow-up chest CTs of patients recovered from COVID-19. [file 12916_2021_2056_MOESM1_ESM.docx]
